# Bioactive, Pro-Apoptotic–Angiotensin Converting Enzyme Inhibitor Effects and Properties of Ultrasound-Treated Traditional Poppy Vinegar Using the Response Surface Methodology Model

**DOI:** 10.1021/acsomega.4c04342

**Published:** 2024-07-29

**Authors:** Seydi Yıkmış, Esra Bozgeyik, Nazan Tokatlı Demirok, Kerem İlaslan, Rana Muhammad Aadil

**Affiliations:** † Department of Food Technology, Tekirdag Namık Kemal University, 59830 Tekirdag, Türkiye; ‡ Vocational School of Health Services, Adiyaman University, 02040 Adiyaman, Türkiye; § Department of Nutrition and Dietetics, Faculty of Health Sciences, Tekirdag Namık Kemal University, 59030 Tekirdag, Türkiye; ∥ Department of Gastronomy and Culinary Arts, School of Applied Sciences, Bahcesehir University, 34000 İstanbul, Turkiye; ⊥ National Institute of Food Science and Technology, University of Agriculture, 38000 Faisalabad, Pakistan

## Abstract

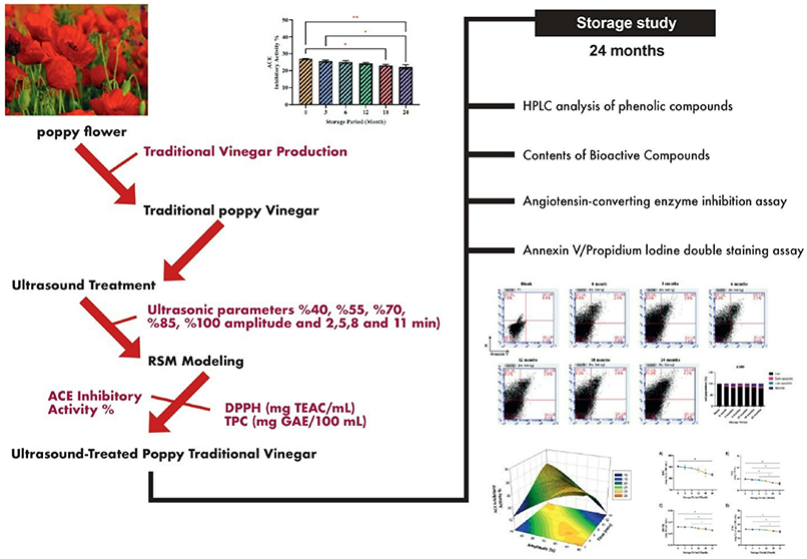

Poppy vinegar with functional properties is a fermented product. This study evaluated traditionally produced poppy vinegar. The study was conducted on poppy vinegar to determine the maximum increase in angiotensin converting enzyme (ACE) inhibitory activity %, total phenolic content (TPC), and radical scavenging activity (DPPH) of the vinegar at different combinations of ultrasound treatment duration (2–14 min) and amplitude (40–100%). The optimal parameters obtained using the response surface methodologies (RSM) were the duration of the ultrasound of 5.5 min and the amplitude of the ultrasound at 57%. When the DPPH values, ACE inhibition %, and TPC and DPPH values obtained with the RSM model were compared with the experimental values, the difference was 9.80, 3.0, and 4.6%, respectively, showing good agreement between actual and predicted values. The higher ultrasound intensities and longer treatment times had a significant effect on antioxidant activity. Poppy vinegar samples significantly induced the apoptosis of lung cancer cells, particularly those stored for 6 and 12 months. The amounts of protocatechuic acid, gallic acid, neohesperidin, hydroxybenzoic acid, resveratrol, rutin, *trans*-cinnamic acid, quercetin, and flavon in poppy vinegar were determined, which decreased significantly as storage time increased. TPC and TFC were determined to be 90.39 mg of GAE/100 mL and 29.86 mg of TEAC/mL, respectively, and there was no significant change in these bioactive compounds after 6 months of storage. The highest value of ACE inhibitory activity was found at the beginning of the storage period. The present study was the first study to examine the bioactive components, ACE inhibition activity, pro-apoptotic activities, and phenolic composition of traditionally produced ultrasound-treated poppy vinegar during storage. The control of production parameters and the design of ideal poppy vinegar fermentation processes could benefit from this research.

## 1 Introduction

Nonthermal technologies preserve nutritional quality, flavor, and overall sensory attributes by providing innovative approaches to food processing and preservation.^1^ Ultrasound is a nonthermal technology which is used in foods.^2,3^ As an environmentally friendly, nonthermal processing technique, ultrasound has excellent potential for use in various food applications.^4^ Ultrasound power ranges from 20 to 100 kHz, with low-intensity ultrasound ranging from 2 to 10 MHz and frequencies from 20 kHz to 500 MHz.^5^ The ability of ultrasound to preserve food’s natural freshness, flavor, and nutritional value while using less energy is making it increasingly popular.^6^ Ultrasound can be used to eliminate enzymes and microorganisms without destroying the nutrients in food as an alternative to thermal treatments.^7^ Health and environmental risks associated with producing carcinogenic halogenated byproducts (using chlorine-based chemical substances) can be reduced using ultrasonic technologies.^8^

Vinegar is made from raw plant materials, and its earliest known use dates back more than 10,000 years.^9^ Beneficial properties of naturally fermented vinegar include antihypertensive, antidiabetic, antitumor, antimicrobial, antioxidant, cholesterol-lowering, and antiobesity.^10^ This research considered vinegar from poppy (*Papaver rhoeas L*.), which belongs to the *Papaveraceae* family.^11^ When crushed, the upright plant grows 20–80 cm tall and produces a pungent odor and white latex.^12^ It is an edible plant used to treat asthma, diarrhea, inflammation, cough, pain, and insomnia.^13^ Due to its abundance of active phytochemicals, mainly phenolic (gallic acid, 3,4-dihydroxybenzoic acid, catechin, caffeic acid, syringic acid, rutin, p-coumaric acid, ferulic acid, resveratrol, quercetin, cinnamic acid, and kaempferol) and other components (calcium, potassium, and sodium), the poppy plant is one of the most widely used plants in the medicinal field.^14−16^

Response surface methodology (RSM) is a set of mathematical and statistical techniques for process design, improvement, and optimization.^9^ A number of recent studies have been carried out on the optimization of ultrasound-treated food products using RSM.^17−21^ Poppy vinegar is a traditional fermented product. There have been studies on the poppy and the poppy sherbet.^22−27^ When the literature was scanned, there were no sources of information about poppy vinegar. This study aimed to improve the ACE inhibition percentage and TPC and DPPH levels of RSM. The contents of total phenolics, flavonoids, ACE inhibitor effects, pro-apoptotic activities, and antioxidant activity in optimized treated poppy vinegar were studied. In addition, the current research provides detailed information about the profile of the phenolic compounds in ultrasound poppy vinegar, which was not reported in previous studies.

## 2 Materials and Methods

### 2.1 Preparation of Poppy Vinegar

The vinegar was made from poppies from Tekirdag, Türkiye. Black areas on the poppy leaves have been eliminated. The poppy leaves have been cleaned and washed, and as described above, the poppy vinegar was produced according to the traditional method.^28^ For ethyl alcohol fermentation, *Saccharomyces cerevisiae* was inoculated at 10^6^ log CFU/mL. For acetic acid fermentation, 5% vinegar was added. The fermentation was maintained at 28 °C for up to 65 days. Acidity (4%) and ethanol content (0.5–1%) were similar. Vinegar mothers formed on the surface of the poppy vinegar when the fermentation finished. Samples of poppy vinegar were stored in sterile glass bottles at −20 ± 1 °C.

### 2.2 Ultrasound Treatment

Ultrasonic treatment was performed in an ultrasonic bath (26 kHz, Hielscher Ultrasonics model UP200 St, Berlin, Germany). The ultrasonic parameters were 40%, 55%, 70%, 85%, and 100% amplitude and 2, 5, 8, and 11 min treatment times in constant mode. After the ultrasonication was finished, the poppy vinegar samples were cooled and stored at −18 ± 1 °C until analyzed (UT-WV).

### 2.3 Experimental Design

The bioactive components of traditional poppy vinegar were optimized by using RSM (Response Surface Methodology). As a process, ultrasound treatment duration (2, 5, 6, 8, 11, and 14 min) and amplitude (40, 55, 70, 85 and 100%) were applied. This led to selecting the central composite and developing 13 experimental designs (Table 1). Lack-of-fit tests, *R*^2^ and adjusted *R*^2^ coefficients, and ANOVA results were considered as the model’s fit values. *X*_1_ (Time) and *X*_2_ (amplitude) were determined as independent variables.
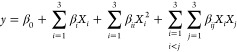
1This formula can be defined as follows: the coefficient of the first order (linear) equation (β_*i*_), the coefficient of the two-factor cross-interaction (β_*ij*_), the coefficient of the quadratic equation (β*_ii_*), the dependent factor (*y*), the intercept term (β_o_), and the independent factors *X*_*i*_ and *X*_*j*_.

**Table 1:** Gene-Specific Primer Sequences

Genes	Primer name	Sequences (5′—3′)
BAX	BAX-Fw	GTCGCCCTTTTCTACTTTGCC
	BAX-Rv	TGGTCACGGTCCAACCACC
BCL2	BCL2-Fw	ATAACGGAGGCTGGGATGC
	BCL2-Rv	TCACTTGTGGCCCAGATAGG
CASP3	CASP3-Fw	CTCTGGTTTTCGGTGGGTGT
	CASP3-Rv	TGAGGTTTGCTGCATCGACA
CASP9	CASP9-Fw	TCAGGCCCCATATGATCGAG
	CASP9-Rv	ACCATGAAATGCAGCGAGGA
GAPDH	GAPDH-Fw	GATCATCAGCAATGCCTCCT
	GAPDH-Rv	TGTGGTCATGAGTCCTTCCA

### 2.4 Contents of Bioactive Compounds

The result for total phenolic content (TPC) was determined by the Folin–Ciocalteau method.^29^ Aliquots of 50 μL, 450 μL, and 2.5 mL of poppy vinegar, distilled water, and reagent (Folin-Ciocalteu) were combined. Saturated sodium carbonate (2 mL) was added following a 5 min dark period. A spectrophotometer (SP-UV/vis-300SRB, Spectrum Instruments, Melbourne, Australia) was then used to measure the absorbance at 765 nm. The results are expressed in milligrams of gallic acid equivalent per 100 g. According to the study by Zhishen et al., the total flavonoid content of watermelon vinegar was determined by the colorimetric method.^30^ After preprocessing, the results of the absorbance measurements were read at 510 nm. The results were expressed as mg of catechin equivalents (CE)/L. The methods of Grajeda–Iglesias et al. (2016) were used to determine the free radical (DPPH) scavenging activity.^31^ The pH-differential method determined the total amount of monomeric anthocyanins (TAC).^32,33^

### 2.5 Analysis of Phenolic Compounds

Chromatography was performed according to Portu et al. (2016); the column used was an Agilent C-18 Age Generix column with dimensions of 250 × 4.6 mm and a packing of 5 μm.^34^ The analysis of polyphenols was performed on an Agilent 1260 chromatograph equipped with a DAD. Solutions A and B were water with 0.1% phosphoric acid and acetonitrile. The following gradient was used: 17% B (0 min), 15% (7 min), 20% (20 min), 24% (25 min), 30% (28 min), 40% (30 min), 50% (32 min), 70% (36 min), and 17% (40 min). The flow rate was 0.80 mL/min at a fixed column temperature of 30 °C. For gradient elution, eluents A and B were used. Solutions A and B were water containing 0.1% phosphoric acid and acetonitrile. The analysis was performed at 280, 320, and 360 nm with a UV–vis spectrophotometer. Values are given in μg/mL.

### 2.6 Inhibition of Angiotensin-Converting Enzyme Assay

For the determination of ACE inhibitory activity, the reaction mixture contained 50 μL of 8 mM HHL, 50 μL of the sample solution, and 100 μL of ACE solution (2.5 mU/mL) as a substrate (incubation for 90 min at 37 °C). The reaction was terminated by adding 250 μL of 1 N HCl. In an aqueduct, hippuric acid was redissolved. The absorbance of the sample of vinegar was measured by using a UV/vis spectrophotometer (SP-UV/vis-300SRB, Spectrum Instruments, Melbourne, Australia) at 228 nm. The activity of the ACE inhibitor was calculated in the following way:
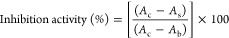
2where *A*_s_ is the absorbance of the reaction mixture (sample), *A*_c_ is the absorbance of the buffer (control), and *A*_b_ is the absorbance when the stop solution has been added before the start of the reaction (blank). The IC_50_ concentration was determined by achieving a 50% reduction in the ACE activity.

### 2.7 Cell Culture

A549 and HTB-54 lung cancer cells were obtained from ATCC (Manassas, VA, USA). Lung cancer cells A549 and HTB-54 were cultivated in DMEM containing 10% FBS. Cells were cultured at 37 °C in an incubator containing 5% CO_2_. Cells that reached the appropriate density were removed with Trypsin for the following experiments, and cell counting was performed.

#### 2.7.1 Annexin V/Propidium Iodine Double Staining Assay

Cells were plated in a 6-well plate at 5 × 10^5^ cells/mL in DMEM containing 10% FBS. The cells were treated with vinegar samples at different concentrations after overnight incubation. Then, cells were collected 24 h after exposure, and the apoptotic cell population was determined using a BD Pharmingen FITC Annexin V Apoptosis Detection Kit I (BD, USA). All steps were carried out following the manufacturer’s recommendations, and readings were performed using the BD Accuri C6 Plus Flow Cytometer (BD Biosciences, USA) device.

#### 2.7.2 Real-Time PCR

A549 and HTB-54 lung cancer cells were seeded in 6-well plates and exposed to different concentrations of vinegar samples. Following 24 h of treatment, the cells were removed, and total RNA was isolated with Trizol. The purity and concentrations of the isolated RNAs were measured spectrophotometrically. Then, cDNA was synthesized from RNA samples with a FIREScript RT cDNA synthesis kit (Solis BioDyne, Estonia), and a 5× HOT FIREPol EvaGreen qPCR Mix Plus kit (Solis BioDyne, Estonia) was used to analyze the expression level of apoptosis-related genes following cDNA synthesis. GAPDH was used as an internal control for gene expression analysis. Primer sequences used in gene expression analysis are shown in Table 1. After each reaction, Ct values at the appropriate threshold were determined, and the gene expression level was analyzed with the 2-ΔCt formula.^35^

### 2.8 Statistical Analysis

The results of the present study were expressed as the mean of the three replicates ± the standard error of the mean. ANOVA (One-way analysis of variance) was carried out. All data were analyzed using the SigmaPlot 12.0 statistical analysis software package (Systat Software, Inc., San Jose, CA, USA) and SPSS 22.0 software package (SPSS Inc., Chicago, IL, USA). Tukey’s test was used to compare the meanings of the poppy vinegar samples.

## 3 Results and Discussion

### 3.1 Optimization of Poppy Vinegar for ACE Inhibition and Bioactive Compounds

Table 2 shows the predicted and experimental results of the ultrasound treatment effects applied to the ACE inhibitory activity, TPC, and DPPH poppy vinegar samples. As a result of the optimization, two independent variables (amplitude and duration) were found in the poppy vinegar. The effects on ACE inhibitory activity % (eq 3), TPC (mg GAE/100 mL) (eq 4), and DPPH (mg TEAC/mL) (eq 5) are shown in the following equations.
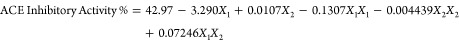
3
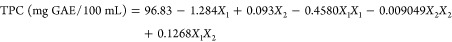
4
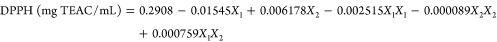
5Table 3 shows the optimization results of the total ACE inhibitor activity, phenolic substance, and DPPH content of poppy vinegar. Table 2 shows the experimental and predicted RSMs’ response to ultrasonic treatment, with vinegar sample results. The effects of independent variables on ACE inhibitor activity, total phenolics, and DPPH were assessed by using RSM modeling.

**Table 2:** Dependent and Independent Factors of RSM Analysis and Results of ACE Inhibitory Activity % and Bioactive Compounds^a^

			Dependent Factors					
	Independent Factors		ACE Inhibitory Activity %		TPC (mg GAE/100 mL)		DPPH (mg TEAC/mL)	
Run no.	*X*_1_ (Time)	*X*_2_(Amplitude)	Experimental data	RSM predicted	Experimental data	RSM predicted	Experimental data	RSM predicted
1	11	55	21.68	21.08	80.39	80.49	0.338	0.336
2	11	85	28.08	27.77	88.02	88.76	0.408	0.408
3	8	70	28.57	28.02	91.48	90.62	0.426	0.427
4	2	70	25.21	25.12	72.86	72.58	0.355	0.352
5	8	70	27.53	28.02	90.83	90.62	0.430	0.427
6	8	70	27.45	28.02	90.55	90.62	0.427	0.427
7	8	70	27.69	28.02	91.23	90.62	0.432	0.427
8	14	70	21.15	21.52	75.94	75.75	0.324	0.322
9	5	85	23.06	23.35	74.97	75.86	0.355	0.357
10	8	100	22.55	22.42	77.47	76.88	0.340	0.333
11	5	55	29.70	29.10	90.16	90.22	0.422	0.418
12	8	70	28.15	28.02	89.90	90.62	0.433	0.427
13	8	40	25.23	25.69	88.02	88.16	0.360	0.362
UT-PV	5.5	57	28.48		90.59		0.428	
Experimental values			26.89 ± 0.33		88.83 ± 0.71		0.409 ± 0.009	
% Difference			9.80		3.0		4.6	

a RSM: Response surface methodology; UT-PV: ultrasound-treated poppy vinegar; ACE: Angiotensin I converting enzyme; TPC: total phenolic content; DDPH: radical scavenging activity; GAE: gallic acid equivalent

**Table 3:** ANOVA Results of ACE Inhibitory Activity and Bioactive Compounds^a^

		ACE Inhibitory Activity %		TPC (mg GAE/100 mL)		DPPH (mg TEAC/mL)	
Source	DF	*F*-Value	*P*-Value	*F*-Value	*P*-Value	*F*-Value	*P*-Value
Model	5	65.66	0.000	111.84	0.000	341.74	0.000
Linear	2	90.41	0.000	21.61	0.000	314.83	0.000
*X*_1_	1	165.28	0.000	42.61	0.000	386.10	0.000
*X*_2_	1	15.54	0.006	0.60	0.000	511.22	0.000
Square	2	71.44	0.000	185.78	0.000	775.38	0.000
*X*_1_*X_1_	1	106.02	0.000	354.57	0.000	908.85	0.000
*X*_2_*X_2_	1	76.46	0.000	86.49	0.000	979.85	0.000
2-Way Interaction	1	142.23	0.000	118.57	0.000	379.54	0.000
*X*_1_*X_2_	1	142.23	0.000	118.57	0.000	379.54	0.000
Error	7						
Lack-of-Fit	3	1.79	0.288	5.41	0.296	1.90	0.272
Pure Error	4						
Total	12						
*R*^2^		97.91%		98.88%		98.89%	
Adj. *R*^2^		96.42%		97.88%		98.09%	
Pred. *R*^2^		86.57%		92.07%		91.92%	

a *X*_1_: time; *X*_2_: amplitude; DF: degrees of freedom; *R*^2^—coefficient of determination; TPC: total phenolic content; ACE: Angiotensin I converting enzyme; DDPH: radical scavenging activity; GAE: gallic acid equivalent; *p* < 0.05, significant differences; *p* < 0.01, very significant differences.

**Figure 1:**
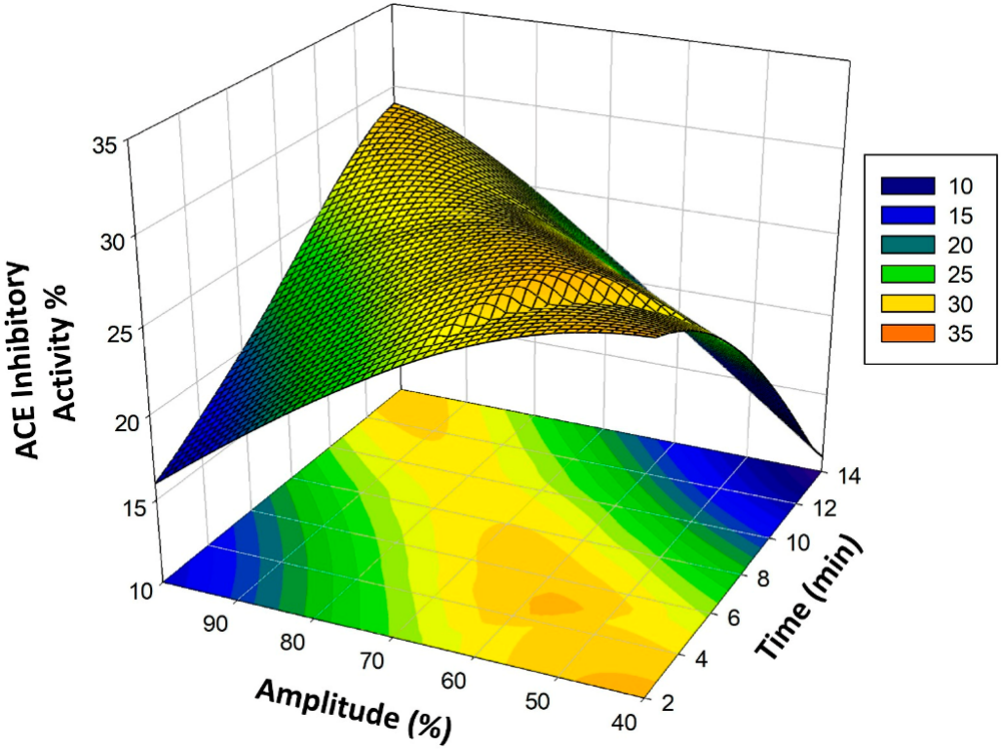
Response surface plots (3D) of ACE inhibitory activity as functions of significant interaction factors.

The optimal levels of the independent variables were determined to be the duration of ultrasound at 5.5 min and the amplitude of ultrasound at 57%. ACE inhibitor activity was 28.48%; DPPH content was 0.428 mg TEAC/mL; and TPC (90.59 mg GAE/100 mL) was obtained under optimal conditions. The UT-PV values from the RSM were compared with those from the repeat test (optimum condition). Consequently, the approximate estimations of ACE inhibitor activity, total phenolic substance, and DPPH results were determined to be 86.57%, 92.07%, and 91.92%, respectively. The activity of the ACE inhibitors was found to increase with time and amplitude. The three-dimensional graph of the modeling (Figure 1) shows the increases. Vinegar has a wide range of physiological functions, including antihypertensive effects, as proven by many studies.^36^ It was found that the interactions (two-way) were statistically significant. The findings were like those outlined by Onyeaka et al. (2023), who saw an increase in total flavonoid, phenolic content, and antioxidant capacity in sonicated samples, with variations in exposure duration and frequency.^5^

**Figure 2:**
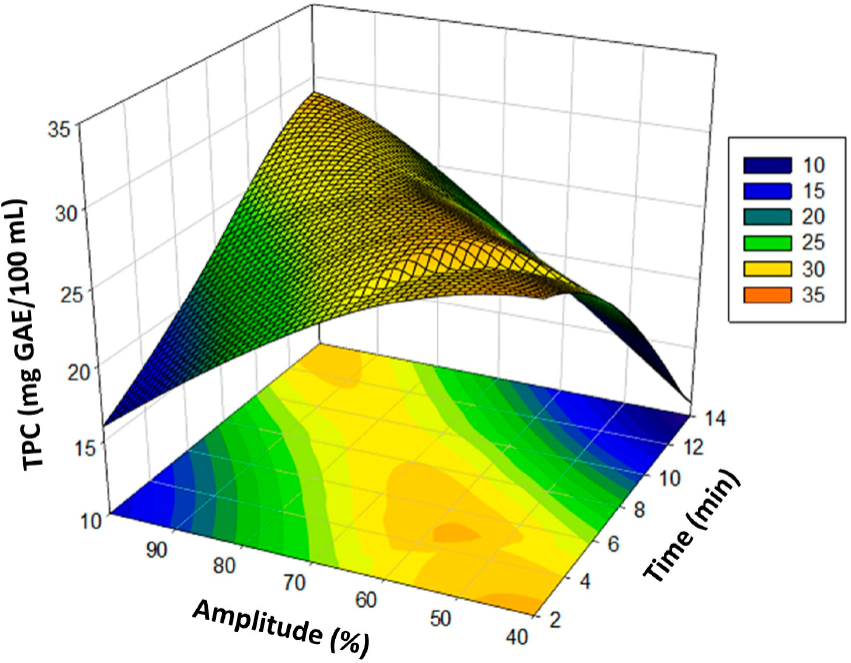
Response surface plots (3D) of TPC (mg GAE/L) as functions of significant interaction factors.

ANOVA for ultrasound-treated poppy vinegar (UT-PV) was significant (*p* < 0.05) with a high coefficient of determination (*R*^2^) of the model for TPC (mg GAE/100 mL) (Table 3). This indicates a high correlation between the experimental and prediction data for the phenolic compound. When examining the effects of amplitude and time, an increase in the amount of TPC (milligrams of GAE/L) was observed. The increases are shown in the three-dimensional graph of the model (Figure 2).

**Figure 3:**
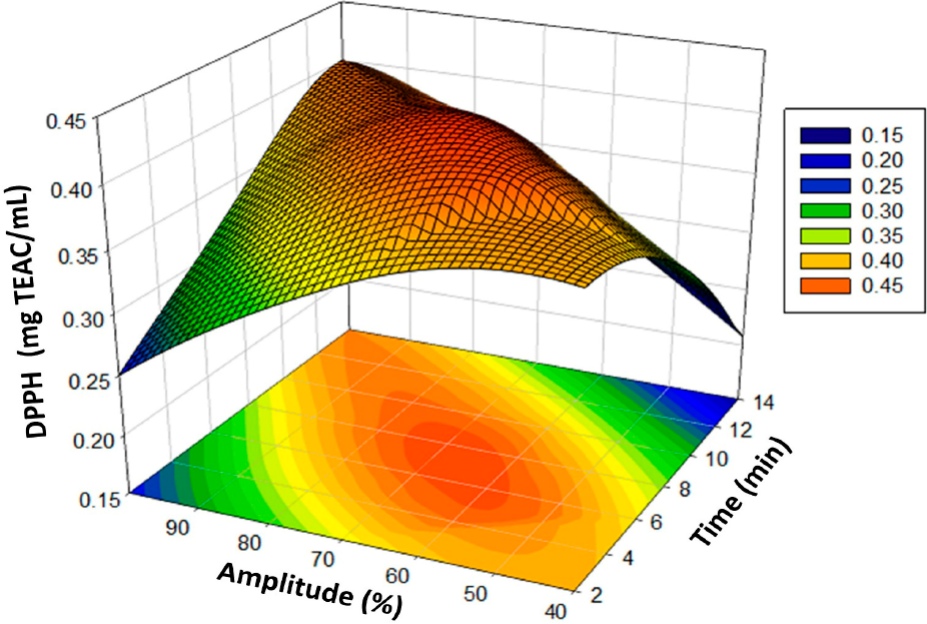
Response surface plots (3D) of DPPH (mg of TEAC/mL) as functions of significant interaction factors.

The linear effects of parameters *X*_1_ and *X*_2_ on the DPPH response (mg TEAC/mL) are significant (*p* < 0.01), as shown by the analysis of variance. An improvement in the DPPH (mg of TEAC/mL) value of the poppy vinegar was observed as the linear effects of the amounts of *X*_1_ and *X*_2_ increased by the formula. These independent quadratic and interaction effects positively influenced the DPPH (mg TEAC/mL) results of poppy vinegar. The increases are shown in the three-dimensional graph of the modeling (Figure 3).

### 3.2 Bioactive Compounds

**Figure 4:**
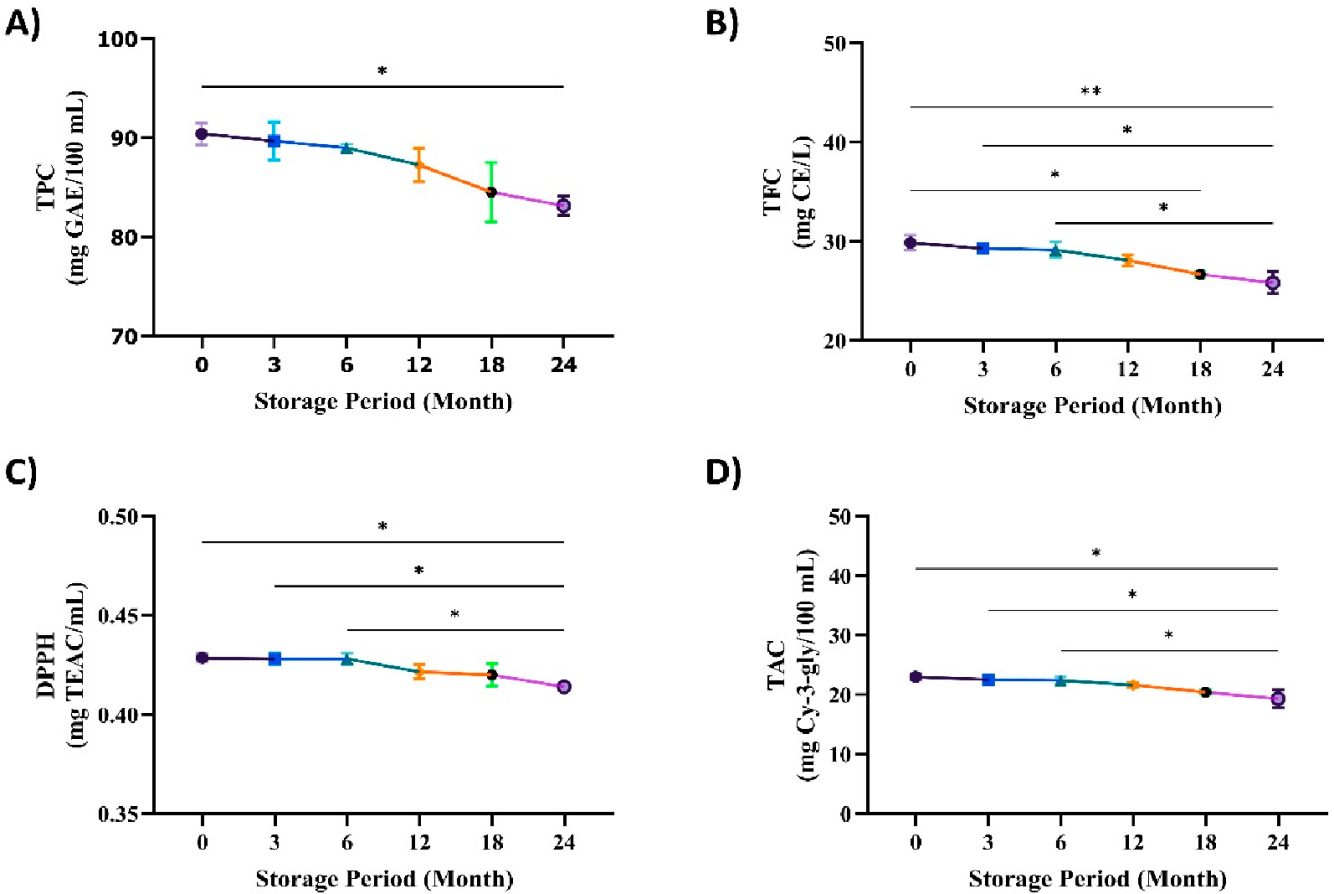
Results for the total phenolic compounds (A), total flavonoid compounds (B), DPPH (C), and total anthocyanin content (D) during the storage of the sample of traditional vinegar from poppy treated with ultrasound. Values with different letters in the rows are significantly different (*p* < 0.05).

Natural antioxidants, including flavonoids and phenolic acids, have received a great deal of attention.^37^ Vinegar’s antioxidant activities are mainly due to its bioactive compounds, including phenolic compounds, phytosterols, and carotenoids.^9^ Ultrasonic cavitation, which involves the bursting and formation of vesicles, facilitates cell wall breakdown and the promotion of the improved extraction of bioactive compounds, such as total phenolics and anthocyanins.^5^ Antioxidant activity was significantly affected at higher ultrasound intensities and longer treatment times (Figure 4). It is well established that in aqueous solutions ultrasound can facilitate the generation of hydroxyl radicals.^38^ Similarly, it was reported that with increased ultrasound intensity and prolonged treatment time the antioxidant activity was progressively reduced in bayberry juice.^39^ In addition, longer storage times and higher storage temperatures cause a decrease in antioxidant activity and anthocyanin content as reported by Dincer et al.^40^ Pearson’s positive correlation coefficients among the bioactive compounds (TFC and TPC) were significantly correlated with DPPH (0.97), gallic acid (0.97), and trans-cinnamic acid (0.99). A similar present study by Hojjatpanah et al.^41^ found that higher phenolic concentrations resulted in lower EC_50_ values. There was no significant change in TPC or TFC after 6 months of storage. However, after 24 months, TPC decreased to 83.14 mg of GAE/100 mL and 25.82 mg of TEAC/mL, decreases of approximately 8.02% and 13.52% compared to the beginning of storage. Similar to the findings of the present study, Grace et al. (2014) found no significant TPC after 4 months of storage; however, after 8 months, TPC dropped to 34.19 mg/g, which is around 14.4% lower.^42^ In addition, Nadeem et al. (2018) reported that the total flavonoid and phenolic content of the ultrasound-treated carrot and grape juice blend was shown to decrease with an increase in storage time of 100 days.^43^

### 3.3 Analysis of Phenolic Compounds

Phenolic compounds are specialized plant metabolites with one or more hydroxyl groups attached to the aromatic ring, ranging from the simplest to the most complex.^44^ Phenolics are responsible for the organoleptic characteristics such as the taste and color of the vinegar.^45^ The contents of ten phenolic compounds (including rutin, gallic acid, trans-cinnamic acid, protocatechuic acid, quercetin, hydroxybenzoic acid, neohesperidin, resveratrol, and flavon) in poppy vinegar were determined (Table 4).

**Table 4:** Properties of Phenolic Compounds of Ultrasound-Treated Poppy Vinegar Optimized for 24 Months of Storage^a^

Phenolic Compounds	Storage Period (Month)					
(μg/mL)	0	3	6	12	18	24
Gallic acid	47.78 ± 0.32^a^	48.00 ± 1.09^a^	46.39 ± 1.03^a^	45.58 ± 0.95^ab^	44.35 ± 1.12^ab^	42.22 ± 0.78^b^
Protocatechuic acid	1.21 ± 0.04^a^	1.13 ± 0.17^a^	1.20 ± 0.05^a^	1.09 ± 0.05^a^	1.12 ± 0.02^a^	1.05 ± 0.04^a^
Catechin	n.d.	n.d.	n.d.	n.d.	n.d.	n.d.
Hydroxybenzoic acid	<0.003	<0.003	<0.003	<0.003	<0.003	n.d.
Vanillic acid	n.d.	n.d.	n.d.	n.d.	n.d.	n.d.
Gentisic acid	n.d.	n.d.	n.d.	n.d.	n.d.	n.d.
p-coumaric acid	n.d.	n.d.	n.d.	n.d.	n.d.	n.d.
Rutin	0.18 ± 0.01^a^	0.18 ± 0.01^a^	0.17 ± 0.00^a^	0.15 ± 0.00^b^	0.15 ± 0.00^b^	0.15 ± 0.00^b^
Ferulic acid	n.d.	n.d.	n.d.	n.d.	n.d.	n.d.
Naringin	n.d.	n.d.	n.d.	n.d.	n.d.	n.d.
o-coumaric acid	n.d.	n.d.	n.d.	n.d.	n.d.	n.d.
Neohesperidin	0.74 ± 0.01^a^	0.73 ± 0.01^ab^	0.71 ± 0.01^abc^	0.70 ± 0.01^abc^	0.69 ± 0.02^bc^	0.67 ± 0.02^c^
Coumarin	n.d.	n.d.	n.d.	n.d.	n.d.	n.d.
Resveratrol	<0.001	<0.001	<0.001	n.d.	n.d.	n.d.
Quercetin	<0.002	<0.002	<0.002	<0.002	n.d.	n.d.
trans-cinnamic acid	0.92 ± 0.00^a^	0.91 ± 0.01^a^	0.89 ± 0.01^ab^	0.82 ± 0.02^bc^	0.75 ± 0.04^cd^	0.73 ± 0.02^d^
Flavon	0.05 ± 0.00^a^	0.05 ± 0.00^a^	0.05 ± 0.00^a^	0.05 ± 0.00^a^	0.04 ± 0.00^b^	0.04 ± 0.00^c^
Total	50.88 ± 0.35^ab^	51.01 ± 1.25^a^	49.42 ± 1.09^ab^	48.38 ± 0.91^abc^	47.10 ± 1.16^bc^	44.85 ± 0.83^c^

a Different letters indicate significant differences among the values within the same row (*p* < 0.05). The results are presented as mean ± standard deviation. n.d.: not detected.

Ten phenolic compounds during 24 months of storage of poppy vinegar after optimization are shown in Table 3. In the UT-PV samples, gallic acid was dominant, and significant reductions were observed during storage. Gallic acid was also determined as one of the main phenolic contents in Tunisian prickly pear vinegar.^45^ A decrease in all phenolic compounds detected was observed after 24 months of storage. This agreed with the results obtained by Aydoğdu et al. (2023), who showed a decrease was observed in the phenolic components of poppy sherbet during storage (30 days), except for ferulic acid and vanillic acid.^26^ Also, similar to the present study, Daei et al. (2023) found a significant decrease in the amount of rutin (from 5.58 μg/g to 2.88 μg/g) in truffle (Terfezia claveryi) vinegar after 160 days of storage.^46^

### 3.4 ACE Inhibitory Activity

**Figure 5:**
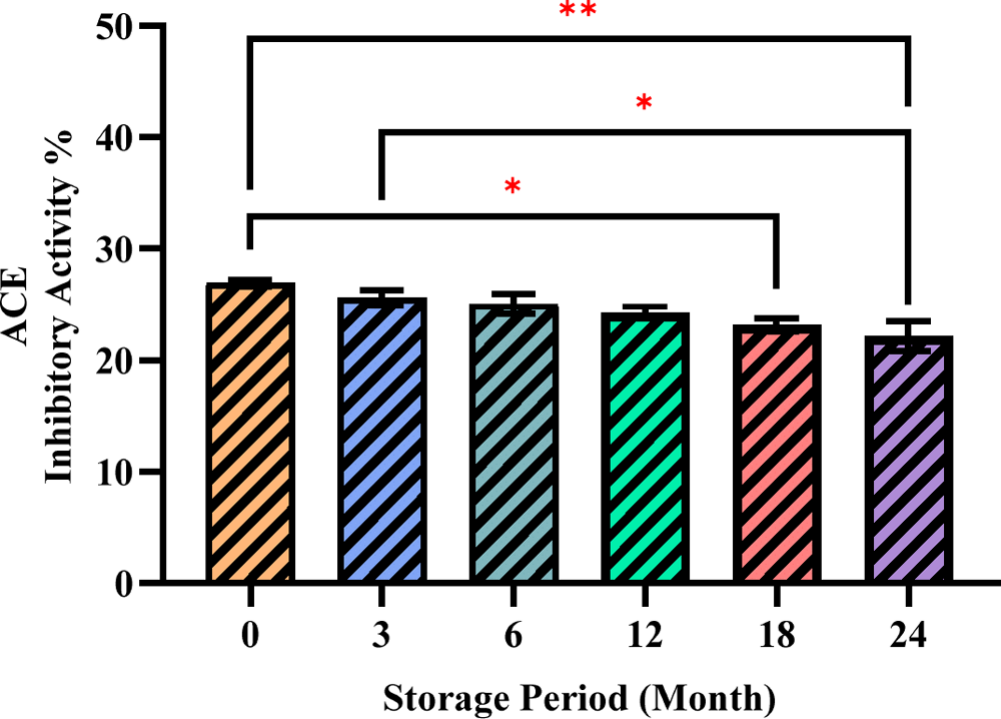
ACE inhibitory activity % of the optimized ultrasound-treated poppy traditional vinegar sample. Statistically significant differences are indicated by letters above bars (ns: no significant; *p < 0.05; (*n* = 3 ± SD).

**Figure 6:**
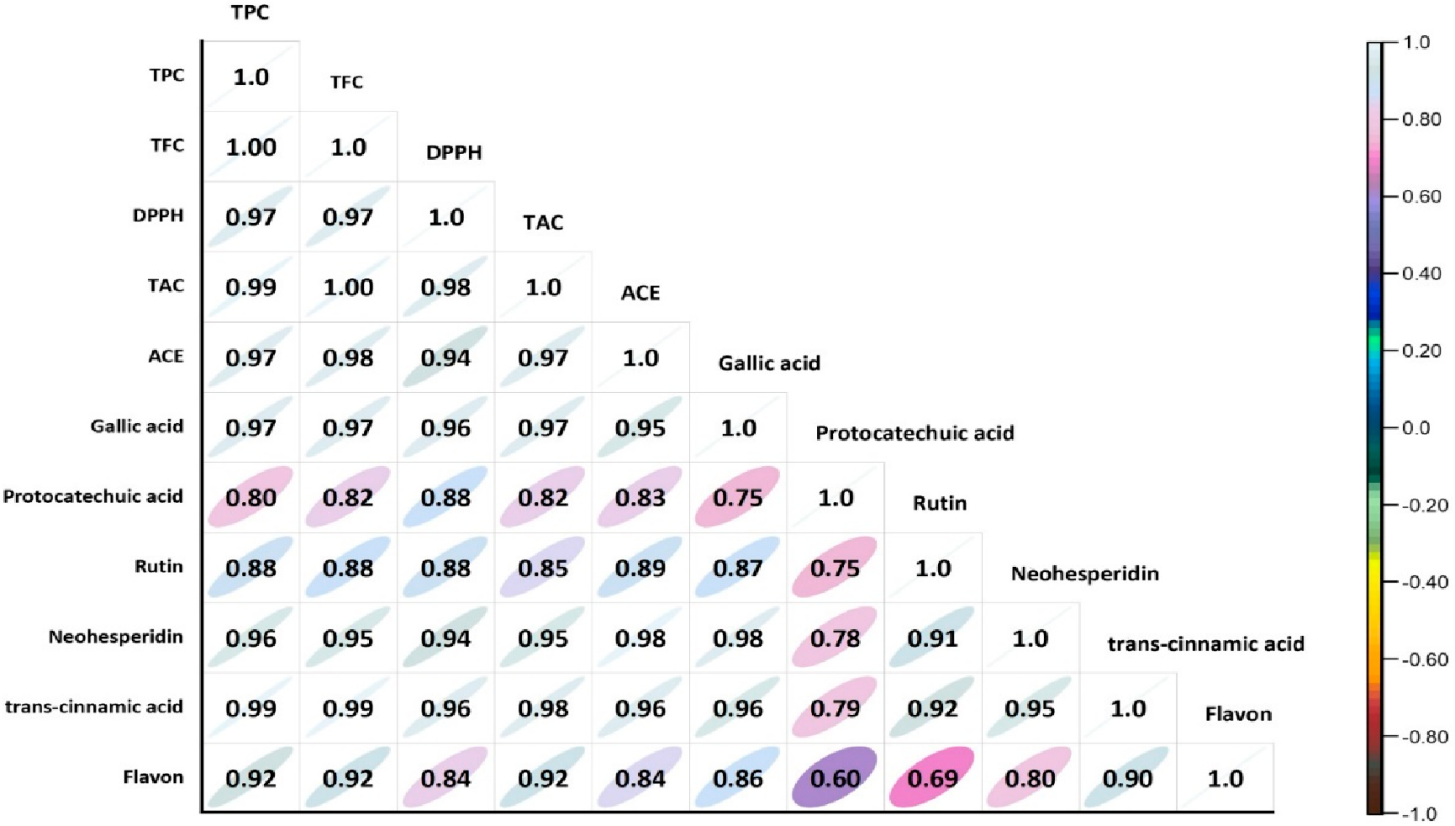
Pearson correlates the coefficients between the antioxidant activity, the phenolic compounds, the bioactive compounds, and the ACE inhibitory activity values of the optimized ultrasound-treated poppy traditional vinegar samples.

Figure 5 shows the ACE inhibitory activity during storage. The highest value was found at the beginning of the storage period. There was a significant decrease in ACE inhibitory activity value after 24 months of storage. Positive correlations were found among ACE with gallic acid (0.95), neohesperidin (0.98), and trans-cinnamic acid (0.96) (Figure 6).

In their study, Kim et al. (2023) found that 10 vinegar-derived acetic acid bacteria isolates (except one) were more effective at inhibiting ACE than antihypertensive drugs. They also found ACE inhibition of 58% and 96.0% at concentrations of 12.5 and 25 mg/mL of fermentation-produced acetic acid, respectively.^47^ According to Wang et al. (2018), the ACE inhibitors in the vinegar egg concentrate (6.7, 8.4, and 10 mg/mL) were 44.9%, 61.0%, and 75.6%, respectively, which were higher than those obtained in our study.^48^ This is thought to be due to the different ACE levels of rice vinegar and eggs. The interest in natural products is growing day by day. Remarkably, vinegar, a naturally fermented product, can have an ACE inhibitory activity.

### 3.5 Apoptosis Stimulating Effect

**Figure 7:**
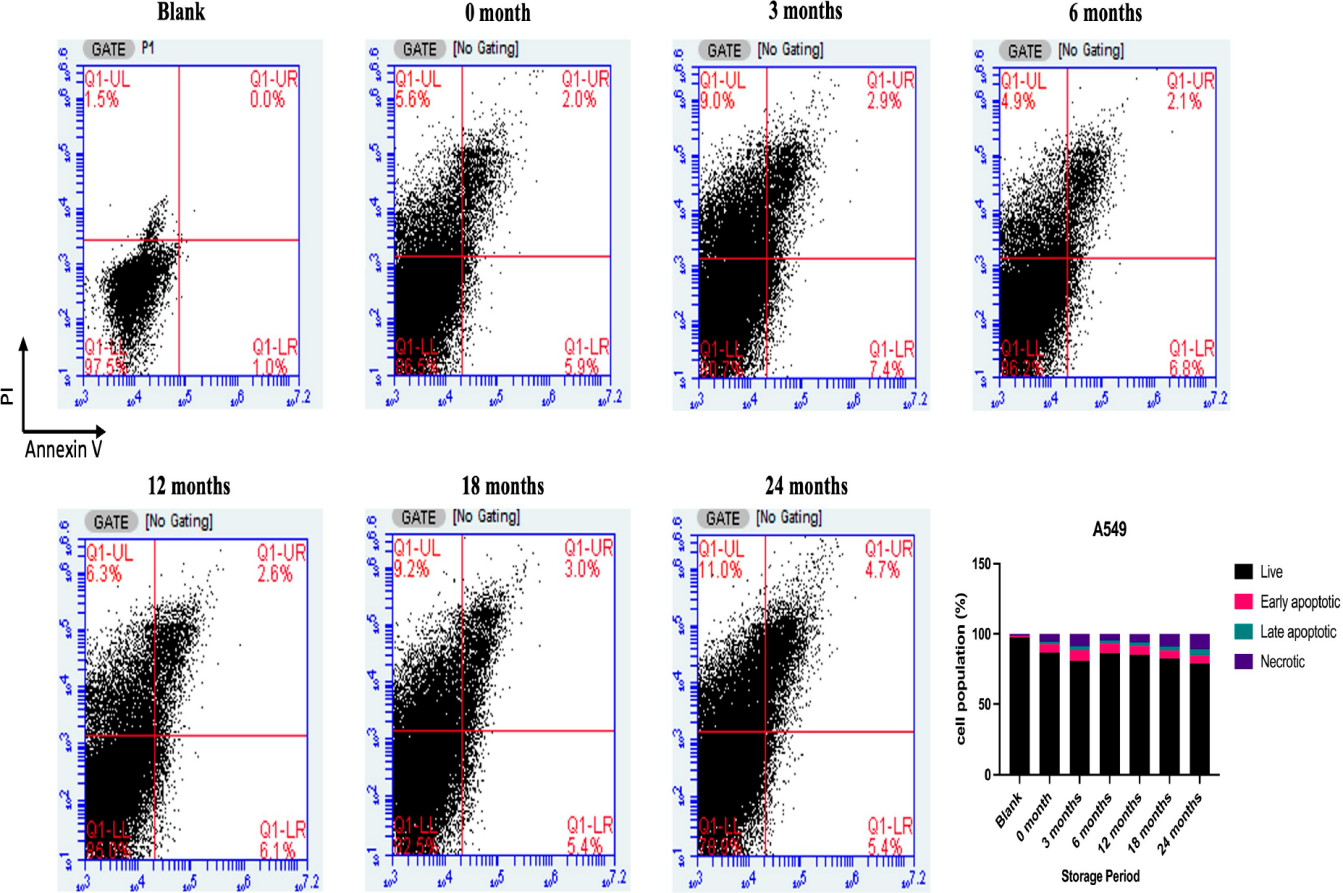
After the exposure of A549 cells to poppy vinegar samples, apoptotic cell populations were stored at different time intervals.

**Figure 8:**
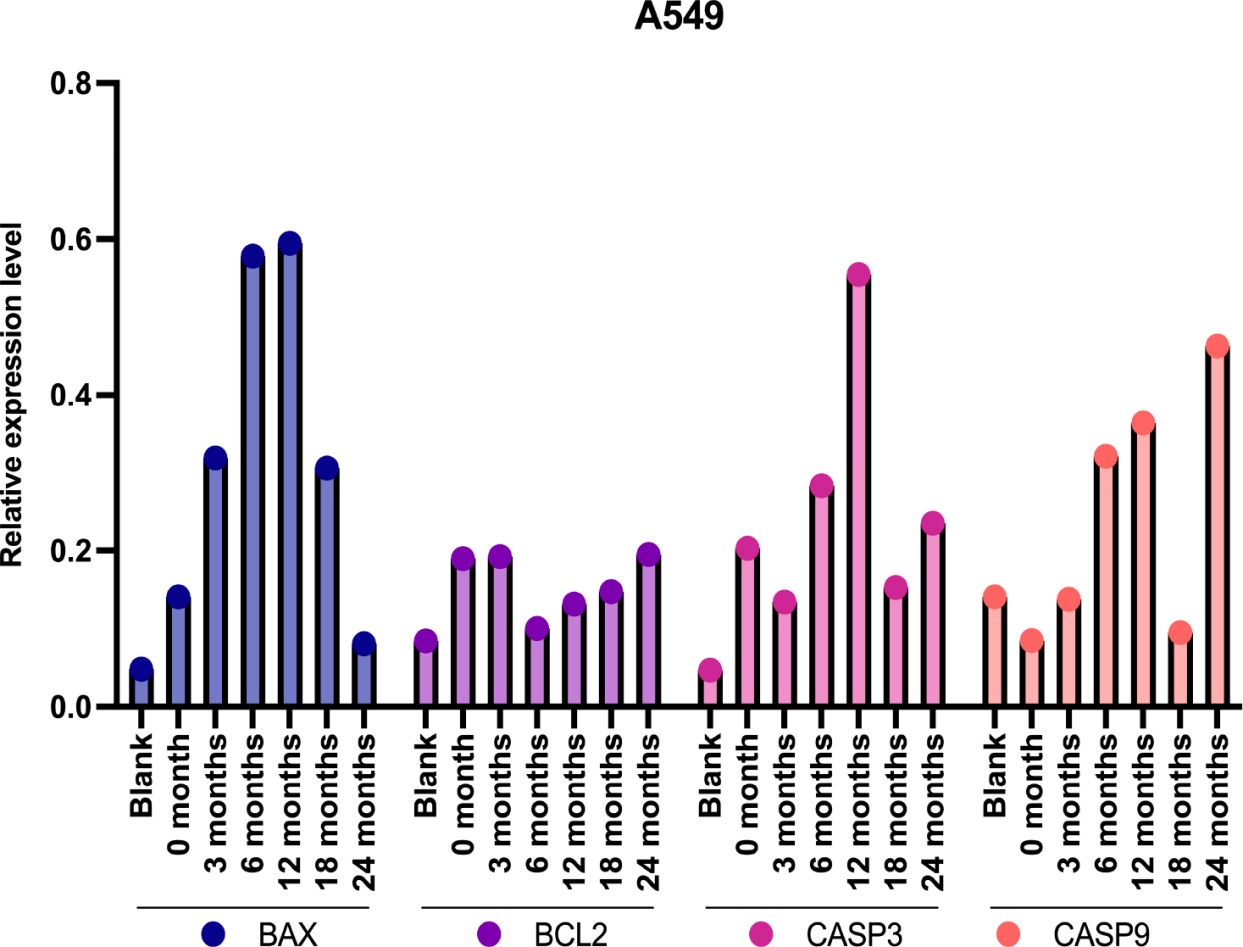
Changes in the expression levels of apoptosis-related genes following exposure of A549 cells to poppy vinegar samples stored at different time intervals.

After 24 h of exposure of A549 cells to 25% poppy vinegar, apoptotic cell populations were analyzed by flow cytometry. It was found that vinegar samples, especially after 6-month and 12-month storage periods, stimulated apoptosis more potently than others (Figure 7). When the expression level of apoptosis-related genes was analyzed, it was determined that the expression level of the pro-apoptotic BAX gene increased compared to the control in A549 cells treated with vinegar samples after 6 and 12 months of storage periods. On the contrary, the expression level of the antiapoptotic BCL2 gene decreased (Figure 8). In addition, the CASP3 gene was found to be highly expressed in A549 cells treated with vinegar samples after 12 months of storage compared to the other groups.

**Figure 9:**
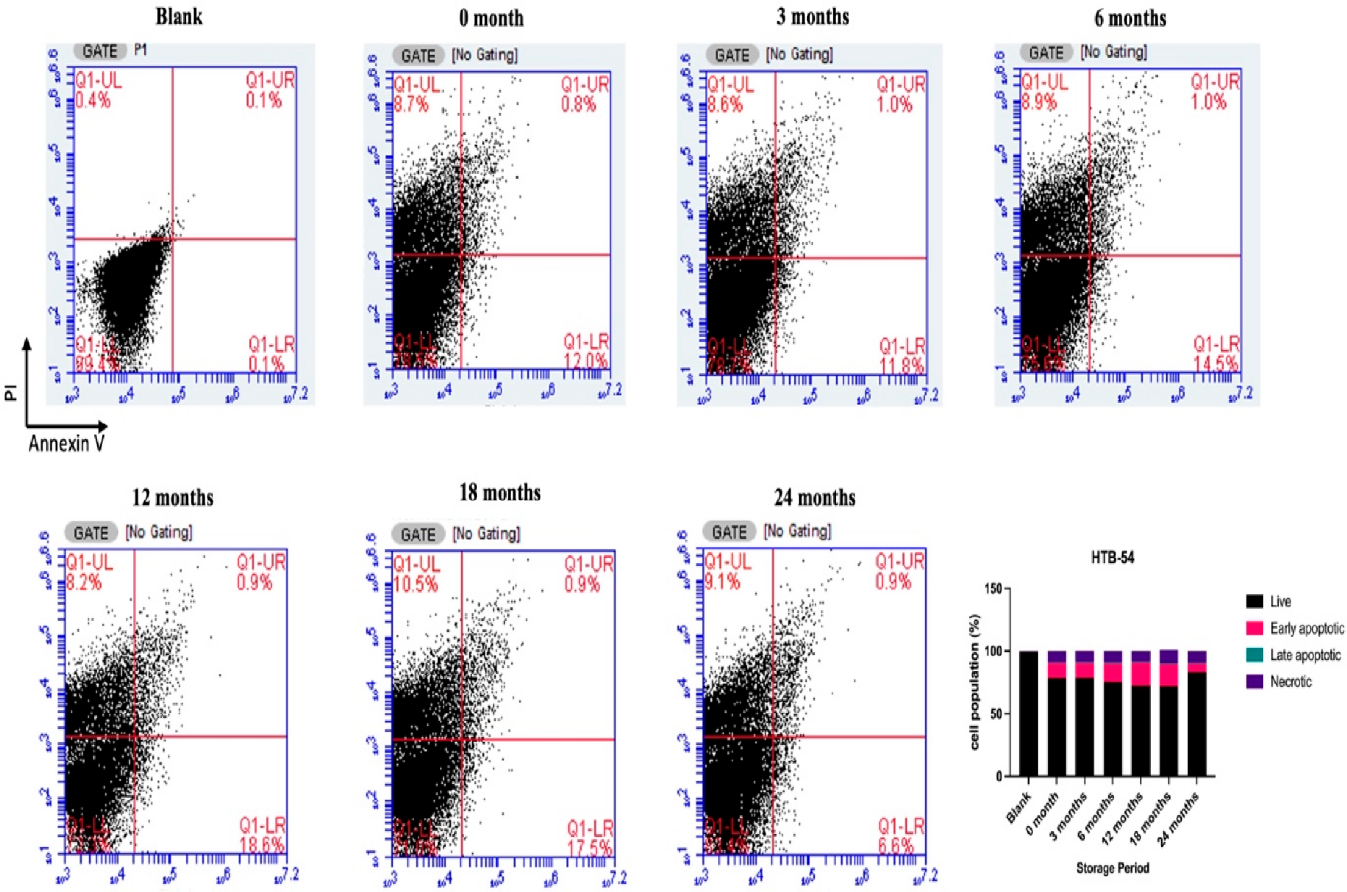
After the exposure of HTB-54 cells to poppy vinegar samples, apoptotic cell populations were stored at different time intervals.

**Figure 10:**
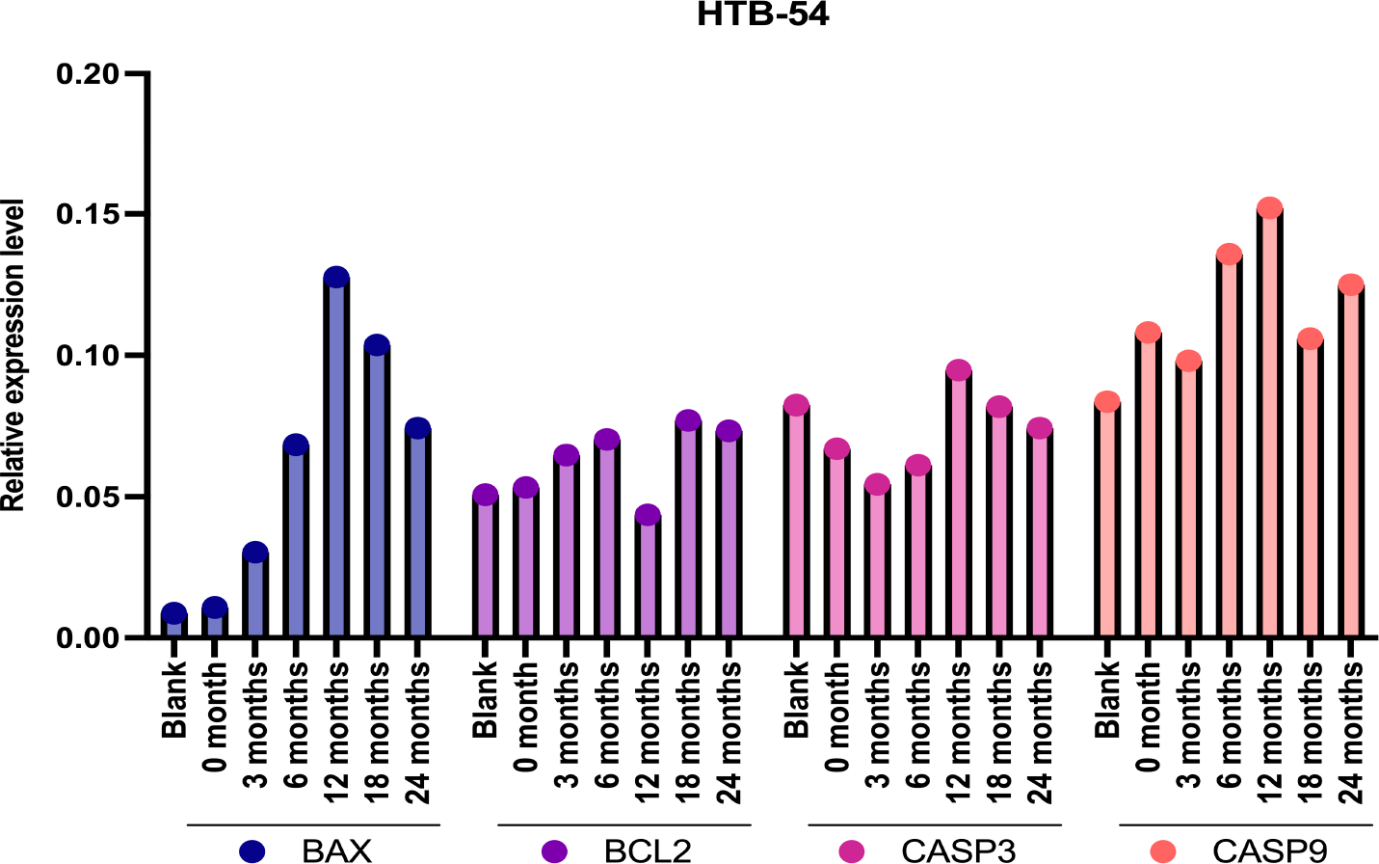
Changes in the expression levels of apoptosis-related genes following the exposure of HTB-54 cells to poppy vinegar samples stored at different time intervals.

On the other hand, in another lung cancer cell line, HTB-54 cells, the apoptotic cell population was higher than the other groups after exposure to poppy vinegar samples stored for 12 months and 18 months (Figure 9). However, considering the increased necrotic cell count in cells exposed to vinegar samples stored for 18 months, it can be argued that the best outcome is observed in the vinegar sample stored for 12 months. In support of these findings, it was observed that exposure of HTB-54 cells to poppy vinegar stored for 12 months for 24 h increased the expression levels of CASP3, BAX, and CASP9 genes, while the expression level of the BCL2 gene decreased (Figure 10).

Vinegar has been used not only as a culinary ingredient since ancient times but also as a folk remedy in the treatment of complex conditions such as cancer and cardiovascular diseases.^49^ In both in vivo and in vitro analyses, it has been reported that vinegar samples produced from various sources exhibit cytotoxic effects on cancer cells.^50−52^ Our study observed that poppy vinegar samples induced apoptosis in A549 and HTB-54 lung cancer cells. It was found that vinegar samples, particularly those stored for 6 and 12 months, had a more significant effect than the others. This observation may be associated with a decrease in the content of the phenolic compounds over time.

## 4 Conclusions

Naturally, fermented products are becoming increasingly popular. An important example of this is traditionally produced vinegar. This is the first study where RSM has been used to optimize the ACE inhibition %, TPC, and TFC of ultrasound-treated poppy vinegar. The bioactive compounds showed no significant change after 6 months of storage. Antioxidant activity was significantly affected by higher ultrasound intensities and longer treatment times. Gallic acid was the main phenolic content in optimized ultrasonically treated poppy vinegar, which decreased significantly after 24 months of storage. The highest level of ACE inhibition was found at the beginning of the storage period. Moreover, poppy vinegar samples stored for 6 and 12 months were found to induce the apoptosis of lung cancer cells in vitro. However, under prolonged storage conditions, the apoptotic effect of vinegar samples was found to be decreased, which can be caused by the reduction of the phenolic content of these samples over time. Given these results, in vivo studies are recommended for future studies.
